# Integrated Role of Arginine Vasotocin in the Control of Spermatogenesis in Zebrafish

**DOI:** 10.3390/ijms262411938

**Published:** 2025-12-11

**Authors:** Maya Zanardini, Hamid R. Habibi

**Affiliations:** Department of Biological Sciences, University of Calgary, 2500 University Drive NW, Calgary, AB T2N 1N4, Canada; maya.zanardini@ucalgary.ca

**Keywords:** arginine vasotocin, AVT, reproductive physiology, in vivo, spermatogenesis, paracrine hormonal regulation

## Abstract

Arginine vasotocin (AVT) has recently emerged as a local regulator of testicular function in fish. Using ex vivo culture system, it was demonstrated that AVT directly stimulates androgen-dependent basal spermatogenesis in zebrafish. In the presence of gonadotropins, AVT enhanced FSH-induced development of early phases of spermatogonial proliferation while blocking FSH-mediated spermiogenesis. However, AVT promoted proliferation of LH-induced pre-meiotic and meiotic germ cell populations without affecting the final stages of spermiogenesis. These findings led to the hypothesis that AVT plays a role by promoting early germ cell proliferation and differentiation while simultaneously inhibiting premature progression through spermiogenesis. To test this hypothesis, we investigated the chronic effects of AVT on adult zebrafish testes, in vivo. Prolonged AVT treatment for 21 days led to dose-dependent accumulation of undifferentiated type A spermatogonia and reduced post-meiotic germ cells and spermatozoa. We also observed decreased plasma 11-ketotestosterone (11-KT) levels and downregulation of *fshr*. This was accompanied by a basal suppression of *avt* and its receptors, *avpr1aa*, *avpr1ab*, *avpr2aa*, *avpr2ab*, *avpr2l*, in both brain and testis during the pre-spawning phase. The present findings, along with those of previously published studies, collectively demonstrate that AVT presence during the early stages of testicular development promotes spermatogonia proliferation while diminishing FSH-induced premature progress toward spermatogenesis. This occurs until later stages, when AVT expression is diminished, allowing for optimal LH-induced spermiogenesis in zebrafish.

## 1. Introduction

The proliferation and differentiation of spermatogonial stem cells (SSCs) to generate mature sperm are tightly regulated processes that remain coordinated throughout the life of an organism. At the systemic level, a multitude of hypothalamic neurohormones, including gonadotropin-releasing hormone (GnRH), stimulate the anterior pituitary to release luteinizing hormone (LH) and follicle-stimulating hormone (FSH), which are primary hormonal drivers of testicular activity and spermatogenesis. In addition to endocrine inputs from the hypothalamic–pituitary–gonadal (HPG) axis, paracrine and autocrine factors produced within the gonad itself provide an additional layer of regulation of germ cell proliferation, differentiation, and maturation [1,2,3,4,5,6,7,8,9,10,11,12].

Several neurohormones classically associated with the brain, including GnRH and gonadotropin-inhibitory hormone (GnIH), are also locally expressed in fish gonads, where they participate in the paracrine and autocrine modulation of steroidogenic and gametogenic functions [1,6,9,13,14,15]. More recently, several studies have demonstrated that arginine vasotocin (AVT), the non-mammalian homolog of vasopressin (AVP), is involved in the local regulation of gonadal activity in fish [16,17,18,19,20,21,22,23,24]. AVT is a phylogenetically conserved nonapeptide with well-known functions in osmoregulation, social behaviour, and reproduction [25,26,27,28,29]. Recent work demonstrated that AVT also affects fish fertility by influencing reproductive behaviour, androgen synthesis, and germ cell development in the testes [19,30,31,32].

Neurohypophysial peptide secretion is highly plastic across vertebrates and varies with reproductive mode. In seasonal breeders, brain and plasma levels of AVT rise during the breeding season and decline during the non-reproductive phases [17,33]. In contrast, non-seasonal species do not exhibit pronounced annual cycles. Instead, AVT secretion is dynamically regulated to support social interactions, reproductive opportunities, and environmental adaptation as needed, rather than being governed by an internal annual calendar [34,35,36,37]. For example, in the African cichlid *Astatotilapia burtoni* and Mozambique tilapia *Oreochromis mossambicus*, *avt* transcript levels in the brain fluctuate with social rank and reproductive readiness [38,39]. Additional work indicates that AVT displays circadian variation but does not follow age-dependent or long-term seasonal profiles in non-seasonal species [36]. Despite these insights, dynamic changes in testicular AVT production throughout reproductive phases in non-seasonal breeders remain unexplored.

In zebrafish, avt and its five G protein-coupled receptor subtypes—avpr1aa, avpr1ab, avpr2aa, avpr2ab, avpr2l—are predominantly expressed in the brain and to a lesser extent in the testis [18]. In the presence of FSH, AVT enhanced FSH-induced early stages of spermatogonial proliferation while restraining FSH-stimulated steroidogenesis and spermiogenesis. Thus, preventing premature spermiation when FSH is required to stimulate spermatogonial development [40]. However, in the presence of LH, AVT did not affect the early stages of spermatogonial development but increased the proliferation of pre-meiotic spermatogonia type B and meiotic germ cell populations, without affecting the final stages of spermiogenesis [40]. Using an ex vivo culture system, it was demonstrated that AVT can stimulate androgen-dependent sperm production while inhibiting spermatogonial proliferation, over 7 days in the zebrafish testis [18]. The action of AVT was blocked in the presence of an androgen antagonist, flutamide, indicating that the production of 11-KT drove the observed stimulatory action of AVT on spermiogenesis. We hypothesized that AVT plays a role by promoting early germ cell proliferation and differentiation while simultaneously inhibiting premature progression through spermiogenesis. We further predict that sustained AVT exposure disrupts spermatogenic progression by limiting spermatogonial activity and thereby reducing germ cell turnover. To address this, we investigated the chronic effects of AVT on testis function in vivo using a combination of histological, stereological, molecular, and biochemical approaches. Our objectives were to: (1) localize avt mRNA and protein within the testis, (2) characterize germ cell composition after long-term AVT treatment, (3) measure plasma 11-ketotestosterone (11-KT) following chronic exposure, and (4) examine the transcript profile of avt and its receptors during pre-spawning phases.

## 2. Results

### 2.1. mRNA and Protein Expression of AVT in the Adult Zebrafish Testis

**Figure 1:**
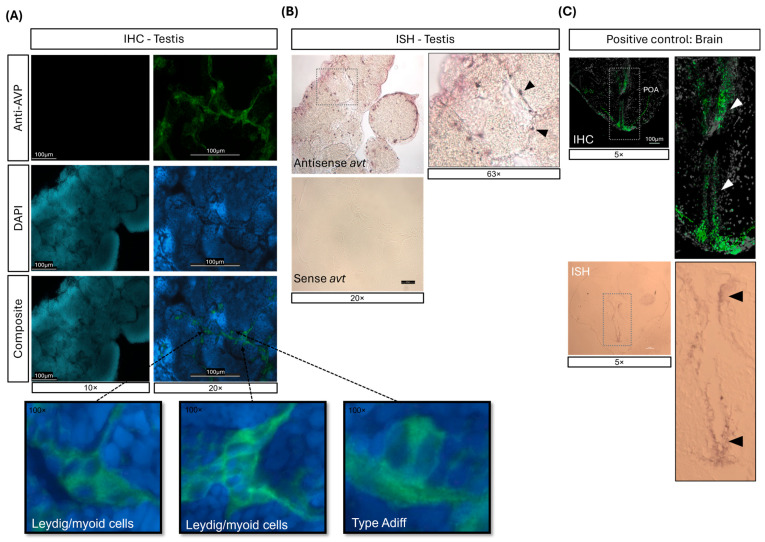
Localization of arginine vasotocin (AVT) expression in adult zebrafish testis detected by (**A**) immunohistochemistry (IHC) and (**B**) in situ hybridization (ISH). (**A**) IHC on testis sections showing AVT-positive staining (top), DAPI nuclear staining (middle) and composite images (bottom). The left panel shows a negative control (no primary antibody), while the right panel shows specific AVT labeling. Inserts highlight cellular localization. (**B**) ISH on testis sections incubated with antisense (top) and sense (bottom) avt RNA probes. Cellular localization of avt is indicated by arrowheads. (**C**) IHC (top) and ISH (bottom) of the forebrain, used as positive control. The arrowheads indicate AVT-positive sites within the preoptic area (POA) of the hypothalamus.

To determine the expression and localization of arginine vasotocin (AVT) in the adult zebrafish testis, we employed in situ hybridization (ISH) and immunohistochemistry (IHC) (Figure 1). Immunofluorescence revealed AVT protein in distinct cell populations located within or adjacent the seminiferous tubules, consistent with Leydig cells in the interstitial compartment and myoid cells surrounding the tubules (Figure 1A). Correspondingly, ISH with an antisense avt probe detected mRNA signals in the same testicular regions (Figure 1B). No signal was observed using the sense probe (Figure 1B) or the IHC negative control lacking primary antibody (Figure 1A, left panel), confirming probe and antibody specificity. Adult male brain sections served as positive controls, with robust AVT mRNA and protein expression observed in the hypothalamic preoptic area (POA), a canonical AVT neuroendocrine center (Figure 1C).

### 2.2. AVT Actions on Spermatogonial Dynamics and Late-Stage Germ Cell Proportions

**Figure 2:**
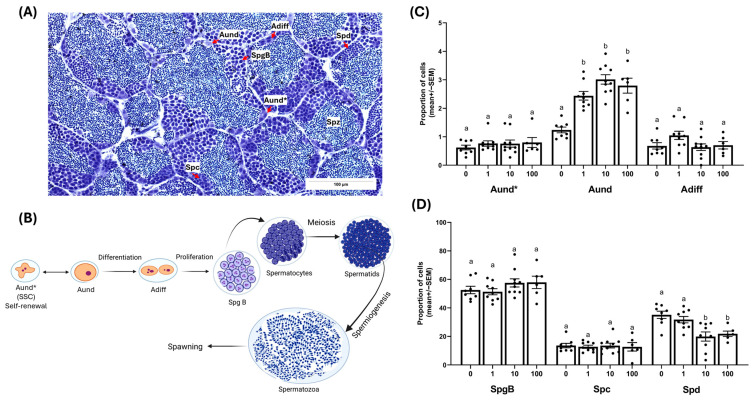
Effects of long-term AVT treatment on spermatogenesis in adult zebrafish testes. (**A**) Representative histological section of adult zebrafish testis, showing the spatial organization and identification of key germ cell types: undifferentiated spermatogonial stem cells (Aund*), undifferentiated spermatogonia (Aund), differentiating spermatogonia (Adiff), type B spermatogonia (SpgB), spermatocytes (Spc), spermatids (Spd), and spermatozoa (Spz). Scale bar = 100 μm. (**B**) Schematic illustration of the spermatogenic process in zebrafish, highlighting the progression from spermatogonial stem cells (Aund*) through differentiation (Aund, Adiff), proliferation (SpgB), meiosis (spermatocytes), spermiogenesis (spermatids), and finally to mature spermatozoa (Spz) released during spawning. (**C**) Bar graphs showing the mean (±SEM) proportions of Aund*, Aund, and Adiff spermatogonia in control (0) and AVT-treated groups (1, 10, and 100 ng/g body weight; *n* = 10 per group). (**D**) Bar graphs showing the mean (±SEM) proportions of type B spermatogonia (SpgB), spermatocytes (Spc), and spermatids (Spd) in the same experimental groups. Different letters above bars indicate statistically significant differences between groups (*p* < 0.05). Dots represent individual data points.

**Figure 4:**
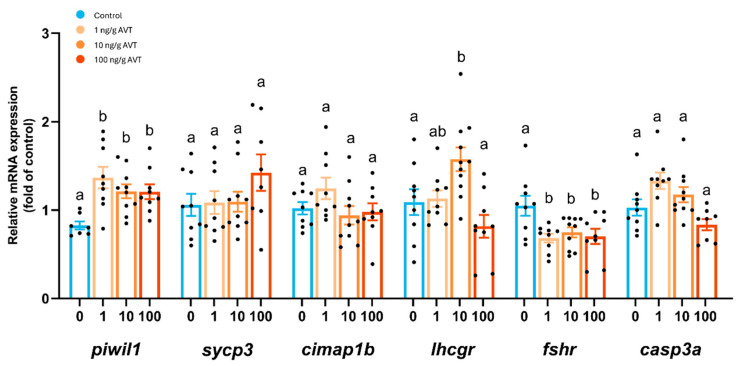
Effects of long-term AVT treatment on the expression of genes involved in spermatogenesis, gonadotropin signaling, and apoptosis in adult zebrafish testis. Relative mRNA expression levels (fold change vs. control) of *piwil1*, *sycp3*, *cimap1b*, *lhcgr*, *fshr*, and *casp3a* in testes from zebrafish treated with 0, 1, 10, or 100 ng/g AVT. Gene expression was normalized to reference genes and expressed as fold change relative to the control group (0 ng/g). Bars represent mean ± SEM; individual data points are shown. Different letters indicate statistically significant differences between groups (*p* < 0.05). Dots represent individual data points.

Long-term administration of AVT at doses of 1, 10, or 100 ng/g body weight did not change the relative abundance of type A spermatogonial stem cells (Aund*) or type A differentiating spermatogonia (Adiff) (Figure 2C). However, results showed an increase in non-stem type A undifferentiated spermatogonia (Aund) in all AVT-treated groups compared to saline controls (Figure 2C). This was supported by elevated *piwil1* transcript levels, a marker for type A spermatogonia, following AVT treatment at all doses (Figure 4). The proportions of type B spermatogonia and spermatocytes (Spc) remained unchanged across all treatments (Figure 2D). Notably, spermatid (Spd) proportions were significantly reduced in fish receiving 10 and 100 ng/g bw AVT compared to controls and the 1 ng/g bw group (Figure 2D). Despite these changes, transcript levels of *sycp3* (spermatocyte marker) and *cimap1b* (spermatid marker) showed no significant differences, as shown in Figure 4.

### 2.3. Spermatozoa Density and Androgen Levels

**Figure 3:**
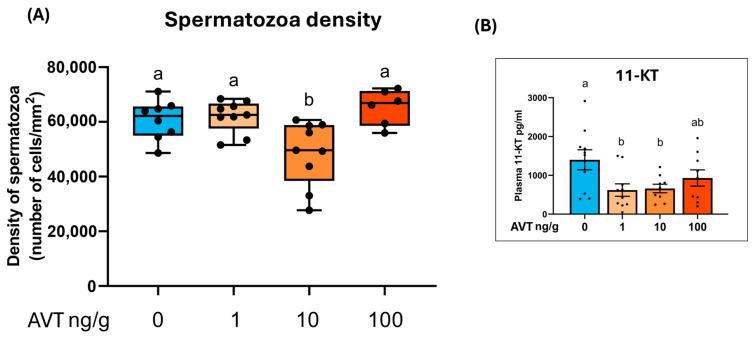
Effect of long-term exposure to AVT on testicular spermatogenesis and plasma androgen levels in adult zebrafish. (**A**) Density of spermatozoa (number of cells/mm^2^) in testis sections from males treated with increasing concentrations of AVT (0, 1, 10, or 100 ng/g body weight). Box plots show median, interquartile range, and individual data points. (**B**) Plasma 11-ketotestosterone (11-KT) concentrations in the same treatment groups. Bars represent mean ± SEM; individual data points are overlaid. Different letters indicate statistically significant differences between groups (*p* < 0.05). Dots represent individual data points.

Spermatozoa density in testicular sections decreased significantly in the 10 ng/g bw AVT group but remained unchanged at 1 and 100 ng/g bw doses (Figure 3A). Plasma 11-ketotestosterone (11-KT), the primary teleost androgen, was significantly reduced in fish treated with 1 and 10 ng/g bw AVT compared to controls but remained unchanged at the 100 ng/g bw dose level (Figure 3B).

### 2.4. Transcript Levels of Gonadotropin Receptors and Apoptotic Signal

To evaluate the effects of AVT treatment on gene expression, we used quantitative PCR (qPCR) to quantify the relative mRNA levels of key genes involved in testicular function, including those related to germ cells, gonadotropin receptors, and apoptosis (Figure 4). The expression of *lhcgr* was significantly increased at 10 ng/g bw AVT, with no changes observed at 1 or 100 ng/g bw. However, *fshr* transcript levels were significantly reduced following treatment with all doses of AVT. Transcript levels of caspase 3a (*casp3a*), an apoptotic marker, remained unchanged across all treatment groups (Figure 4).

### 2.5. Avt and Avt Receptors Transcripts Are Downregulated in the Testis of Pre-Spawning Fish

**Figure 5:**
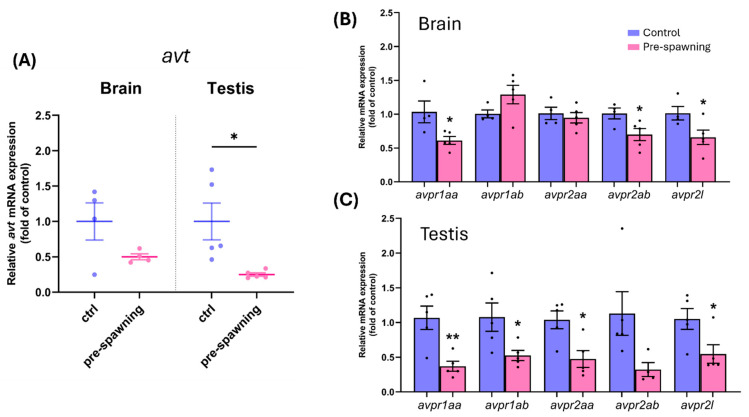
Relative mRNA expression of *avt* and avt receptors in brain and testis of control and pre-spawning zebrafish. (**A**) Relative mRNA expression of avt in brain and testis. (**B**) Expression of AVT receptor subtypes *avpr1aa*, *avpr1ab*, *avpr2aa*, *avpr2ab*, and *avpr2l* in the brain. (**C**) Expression of the same receptor subtypes in the testis. Each point represents an individual sample; data are presented as mean ± SEM. Asterisks indicate statistical significance: * *p* < 0.05 and ** *p* < 0.01. Dots represent individual data points.

To explore the regulation of the AVT system during reproductive cycling, we quantified mRNA levels of *avt* and its receptor subtypes—*avpr1aa*, *avpr1ab*, *avpr2aa*, *avpr2ab*, and *avpr2l*—in the brain and testis of pre-spawning zebrafish. Relative *avt* transcript levels were significantly decreased in both the brain and testis of pre-spawning fish compared to controls (Figure 5A). Specifically, brain *avt* expression was reduced to 0.5 ± 0.04-fold versus 1.0 ± 0.26 in controls, while testicular *avt* levels declined to 0.24 ± 0.02-fold (*p* < 0.05). In the brain, *avpr1aa*, *avpr2ab*, and *avpr2l* transcripts were significantly lower in pre-spawning fish, whereas *avpr1ab* and *avpr2aa* showed no significant change (Figure 5B). In the testis, all vasotocin receptor transcripts were significantly downregulated during pre-spawning except *avpr2ab*, with *avpr1aa* exhibiting the most pronounced reduction (*p* < 0.01) (Figure 5C).

## 3. Discussion

The present study provides the first in vivo evidence that chronic arginine vasotocin (AVT) exposure disrupts spermatogenesis in zebrafish. Immunohistochemistry and in situ hybridization revealed the presence of AVT expression in the interstitial compartment, consistent with localization in peritubular myoid cells, as supported by recent single-cell transcriptomic data [24]. The presence of AVT transcripts and protein in the testis strongly suggests local autocrine/paracrine action. Although circulating levels of AVT in zebrafish are unknown, it has been measured in teleosts, ranging from 1 to 20 fmol mL^−1^ [41]. Thus, while hypothalamic–pituitary endocrine effects may contribute to reproductive physiology, AVT is likely to act locally within gonads to regulate spermatogenesis, aligning with reported gonadal vasotocin/receptor expression in teleosts. Hence, the present study supports the hypothesis that AVT plays an endocrine/paracrine role in the regulation of testicular function, aligning with the reported vasotocin and receptor expression in the gonads of zebrafish and other teleost species [18,31,42].

Previous ex vivo studies indicated that AVT (10 nM) directly stimulated basal 11-KT production and spermiogenesis in zebrafish testes, while simultaneously reducing the proliferation of type B spermatogonia [18]. We predicted that sustained AVT exposure would disrupt spermatogenic progression by limiting spermatogonial activity and thereby reducing germ cell turnover, eventually depleting the pool of spermatids and spermatozoa. To test this hypothesis, we extended the treatment period of AVT in vivo to test the systemic action of the peptide in the zebrafish testis. Adult zebrafish were administered repeated injections of zebrafish-specific AVT at doses consistent with those used in prior ex vivo studies [18]. Histomorphological analysis revealed that chronic AVT administration resulted in a marked increase in type Aund spermatogonia (non-stem), with no changes in the proportions of stem (Aund*) or differentiating (Adiff) germ cell populations. Because Aund* and Aund together form the pool that supports both self-renewal and differentiation, these findings suggest that AVT disrupts the commitment step, leading to an accumulation of type A undifferentiated cells. This effect was confirmed by elevated *piwil1* expression, a marker for type A spermatogonia, across all treatment groups. The developmental block at this stage coincided with a significant reduction in spermatid numbers at intermediate and high AVT doses. However, type B spermatogonia and spermatocytes, along with transcript levels of meiotic and post-meiotic markers (*sycp3* and *cimap1b*), remained unchanged.

Prolonged AVT treatment also influenced sperm output, with a significant reduction in spermatozoa density at 10 ng/g but not at 1 and 100 ng/g, suggesting a non-linear, dose-dependent effect. The latter could result from receptor desensitization or biphasic, dose-dependent actions of AVT of zebrafish testis. Based on the present results, we cannot distinguish between these possibilities. Long-term AVT exposure suppressed plasma 11-KT, thereby impeding androgen-dependent spermatogenic progression—a result that contrasts with our previous finding that short-term AVT application boosts 11-KT secretion and sperm production [18]. The present study further demonstrated that AVT treatment downregulated FSH receptor (*fshr*) transcript levels, likely diminishing Sertoli cell support for germ cell proliferation and thereby reducing premature spermatogenesis. Importantly, no changes in *casp3a* expression were observed, indicating that the reduction in germ cell numbers was unlikely attributable to increased apoptosis but rather to alterations in cell proliferation or differentiation. Taken together, the data indicate a time-dependent action of AVT: acute exposure promotes androgen production and spermiogenesis, whereas prolonged exposure disrupts spermatogonial differentiation and reduces sperm output. It should be noted that we cannot determine if AVT action on germ cells is direct or indirectly mediated via Sertoli/Leydig cells based on the present results. While we demonstrate AVT expression in the testis and brain, the present study tested the actions of exogenous AVT injections. In this context, while this effectively demonstrates AVT’s capability, it does not confirm its physiological necessity.

As previously demonstrated, all five vasotocin receptors are expressed in the zebrafish testis, with distinct distribution patterns across germ and somatic cells. Different AVT receptor subtypes are known to couple to distinct intracellular signaling pathways: avpr1aa, avpr1ab, avpr2aa, and avpr2ab primarily couple to Gq/11 proteins, activating diacylglycerol (DAG), inositol triphosphate (IP3), and Ca^2+^ signaling cascades, whereas avpr2l couples to Gs proteins, stimulating the cAMP/PKA pathway [43]. This differential receptor coupling may underlie divergent physiological effects on germ cell proliferation, differentiation, and steroidogenesis. Despite this, the downstream pathways linking AVT to germ cell fate, fshr downregulation, and 11-KT suppression remain unresolved. The present study did not investigate the functions of receptor subtypes coupled with distinct physiological processes. Future studies employing receptor-selective pharmacology and genetic loss-of-function approaches will be necessary to elucidate the specific roles of each AVT receptor subtype in zebrafish testicular physiology.

The dynamic regulation of the AVT system further emphasizes its relevance to reproductive readiness. Immediately before spawning, transcript levels of *avt* and its receptors (especially *avpr1aa* and *avpr2l*) were significantly decreased in both brain and testis, suggesting that this coordinated downregulation helps synchronize neuroendocrine and local testicular signalling and prevent excessive AVT activity during critical reproductive windows. Evidence from mammalian studies suggests that high concentrations of vasopressin can have a detrimental effect on sperm function, fertilization, and even embryo development in mice [44]. Studies using V1a receptor-deficient mice and oxytocin receptor (OXTR) antagonists have demonstrated that the V1a receptor inhibits sperm maturation, resulting in increased motility and hyperactivation in its absence. Conversely, OXTR promotes sperm maturation, with its inhibition reducing hyperactivation [45]. These findings underscore that although neurohypophysial peptides have conserved roles in male gametogenesis, the consequences of acute versus chronic AVT exposure are species- and context-specific, highlighting the need for further comparative analysis.

## 4. Materials and Methods

### 4.1. Zebrafish

Adult male zebrafish (*Danio rerio*) were kept under standard laboratory conditions in partially recirculating aquaria, maintained at 28 ± 1 °C with a 14:10 h light/dark cycle, pH 7.6, and conductivity between 750 and 800 μS. The fish were fed twice daily with a commercial diet (Gemma Micro 500, Skretting, Westbrook, ME, USA) supplemented by live brine shrimp (*Artemia salina*). All experimental procedures complied with the University of Calgary Animal Care Committee guidelines (protocol # AC24-0042).

### 4.2. Arginine Vasotocin

We used synthetic zebrafish arginine vasotocin (AVT), purchased from ChinaPeptides Co., Ltd. (Shanghai, China), which is the endogenous nonapeptide hormone of *Danio rerio*, with sequence CYIQNCPRG containing a disulfide bridge between Cys1 and Cys6 (Molecular Weight: 1051.22 g/mol; peptide purity >95%). The peptide was dissolved and aliquoted in 1× PBS, aliquoted, and stored at −20 °C until use.

### 4.3. Intraperitoneal (i.p.) Injections, In Vivo

To investigate the long-term effects of arginine vasotocin on testicular function, adult fish were randomly assigned to four treatment groups: control (saline), 1 ng/g body weight (BW) AVT, 10 ng/g BW AVT, and 100 ng/g BW AVT (*n* = 20, 15 males and 5 females per group). Treatments were administered to anesthetized fish via intraperitoneal (i.p.) injection every 3 days over 21 days (i.e., on days 1, 4, 7, 10, 13, 16, and 19). The AVT doses were prepared freshly in 1× PBS (saline) and adjusted to deliver the appropriate concentration per gram of BW, using a Hamilton syringe. At the end of the 21-day treatment period, fish were anesthetized and euthanized for tissue collection. Blood was collected using a heparinized micropipette tip by transecting the caudal fin, following the procedure described by Pedroso (2012) [46]. The collected blood was transferred into a 0.5 mL Eppendorf tube and centrifuged at maximum speed (typically 13,000× *g*) for 15 min at 4 °C. The resulting plasma was aspirated into a new tube and stored at –20 °C for steroid analysis. Testes were dissected and either fixed overnight in 1.6% paraformaldehyde, 2.5% glutaraldehyde, and PBS 1X (pH: 7.2) for histological examination or snap-frozen in liquid nitrogen and stored at –80 °C for gene expression analysis.

### 4.4. Pre-Spawning Experimental Design

To investigate changes in the transcript levels of *avt* and its receptors in pre-spawning zebrafish, two experimental groups were established: Control and Pre-spawning. The Control group consisted of five breeding tanks, each containing a single adult male without female presence, to characterize the transcript levels of avt and its receptors under non-spawning conditions. In this context, the physical presence of males and females is necessary to facilitate visual, chemical, or physical interactions that are necessary to initiate spawning. By excluding female cues, the control provides a reference for “non-reproductive stimulation” in males.

The Pre-spawning group consisted of five breeding tanks, each housing one male and two females (1:2 male-to-female ratio; total of 5 males and 10 females) [47]. In the pre-spawning group, males and females were separated overnight using a transparent plastic divider within each breeding tank, allowing social, visual and chemical communication without physical contact [48]. In the morning, the dividers were removed, allowing male and female fish to interact physically until they displayed clear signs of courtship behavior as described by Darrow (2004) [49]. This brief interaction further stimulates the fish and prepares them, both physiologically and behaviorally, to spawn once contact with females is complete. However, immediately following this short mating exposure, but before spawning, fish were euthanized to collect testis and brain samples. The entire experiment was replicated three times.

### 4.5. Histology and Germ Cell Quantification

Histological analysis was conducted following established methods [14,18]. Briefly, fixed testicular samples were dehydrated through a graded ethanol series, embedded in Technovit 7100 (Heraeus Kulzer, Wehrheim, Germany), and sectioned at 3 μm thickness. Sections were stained with 0.1% toluidine blue in 1% sodium borate. Testicular morphology and germ cell populations were examined under a light microscope. For each sample, five bright-field images were captured, and germ cells were quantified within a 20,000 µm^2^ area, classifying cells by developmental stages including spermatogonia types Aund* (stem cells), Aund (non-stem), Adiff, type B spermatogonia, spermatocytes, and spermatids. The counts were expressed as relative proportions. Spermatozoa were quantified by automated counting using Fiji (ImageJ) software (Version 1.44p) [50] over a total area of approximately 0.17 mm^2^ (517.56 µm × 327.75 µm), and the results were normalized to the total tissue area—excluding non-tissue regions—and expressed as spermatozoa density per mm^2^ as described previously [18].

### 4.6. Testicular Localization of Avt by In Situ Hybridization (ISH)

The expression sites of avt mRNA in the zebrafish testis were identified by in situ hybridization, adapting the protocol from Thisse and Thisse [51]. Digoxigenin-labeled sense and antisense cRNA were synthesized from specific PCR products generated with primers containing T7 RNA polymerase promoter sequences (5′TAATACGACTCACTATAGGG-3′) at 5′-ends (sequences are shown in Table 1). PCR product was extracted, confirmed by sequencing, purified with PureLink™ Quick Gel Extraction and PCR Purification Combo Kit (Invitrogen, Cat. K220001, Thermo Fisher Scientific, Waltham, MA, USA) and transcribed using DIG-UTP (digoxigenin) and RNA T7 polymerase Roche kit (Roche 11758888001, Roche, Basel, Switzerland). Fresh tissues from non-treated fish were fixed in 4% paraformaldehyde overnight in RNase-free conditions, dehydrated, cleared in xylene, embedded in paraffin (Paraplast^®^, Sigma, Merck KGaA, Darmstadt, Germany), and sectioned with a 5 μm thickness. Subsequently, the slides were rehydrated with Et-OH washes. Slides were then treated with proteinase K (10 μg/mL) at 37 °C for 20 min and incubated with a hybridization solution containing either sense or antisense RNA probe at 70 °C overnight. The day after, slides were washed, blocked for 1 h at RT, and incubated with the anti-DIG-AP primary antibody (anti-digoxigenin-alkaline phosphatase conjugated) diluted 1:2000 in the blocking solution at 4 °C overnight. Tissues were then washed to remove excess antibody and incubated with staining solution NBT/BCIP (nitro-blue tetrazolium chloride/5-Bromo-4-chloro-3′-indolyphosphate p-toluidine salt) (Thermo Scientific Pierce, Thermo Fisher Scientific, Waltham, MA, USA). Slides were photographed using the microscope Leica DM6000 B (Leica Microsystems, Wetzlar, Germany).

### 4.7. Localization of Testicular Avt-Expressing Cells by Immunohistochemistry (IHC)

Immunohistochemistry (IHC) was performed as previously described [52] to determine the presence and location of AVT-expressing cells in the zebrafish testis. Briefly, testes explants were fixed in 4% PFA for 24 h at 4 °C. Following fixation, the tissue was washed in 1× PBS, incubated in 30% sucrose in PBT (PBS with 0.1% Triton X-100) overnight, embedded in Neg-50™ Frozen Section Medium (Fisher Scientific, Toronto, ON, Canada), and frozen at −80 °C until sectioning. Sections of 10 µm thickness were cryosectioned, placed on Superfrost Plus slides (Fisher Scientific, ON, Canada), and stored at −80 °C. Slides were then washed for 30 min in 1% PBT and incubated for 1 h in blocking buffer (1% BSA in PBS, 0.05% Tween, 0.05% normal goat serum). Slides were incubated in a humidified dark chamber at 4 °C overnight with primary antibody anti-AVT (rabbit, Immunostar, Hudson, WI, USA, cat# 20069) diluted in the blocking buffer 1:250. It is worth noting that the anti-AVP antibody used in the present study was previously characterized for specificity and use in zebrafish [53]. The following day, slides were washed 4 × 10 min with 0.1% PBT and incubated for 2 h at room temperature with the secondary antibody Goat Anti-Rabbit IgG H&L Alexa Fluor^®^ 488 diluted 1:500 (cat# ab150077 Abcam). After 2 × 10 min washing with PBT, nuclei were counterstained with DAPI, slides were mounted and stored at 4 °C. Images were acquired using a Leica DM6000 B microscope equipped with a DFC550 fluorescence camera.

### 4.8. Quantitative Real-Time PCR (qPCR)

Molecular analyses were carried out as previously described [18]. Total RNA was isolated from testicular tissue using TRIzol reagent (Invitrogen, Toronto, ON, Canada) according to the manufacturer’s instructions [54]. Complementary DNA (cDNA) synthesis was conducted using 1 µg of RNA with the iScript™ cDNA Synthesis Kit (Bio-Rad, Mississauga, ON, Canada). Quantitative PCR (qPCR) was carried out using gene-specific primers targeting steroidogenesis-related genes, germ cell markers, and vasotocin receptors. Expression levels were normalized to the internal reference gene *eef1a1l1* (*elongation factor 1 alpha 1-like 1*), and relative quantification was determined using the 2^^−ΔΔCt^ method. Primer sequences were derived from previous studies [18].

### 4.9. Quantification of Plasma 11-Ketotestosterone (ELISA)

Plasma concentrations of 11-ketotestosterone were determined using a commercial enzyme-linked immunosorbent assay (ELISA) kit from Cayman Chemicals (Ann Arbor, MI, USA), following the instructions provided by the manufacturer. Absorbance measurements were obtained at 412 nm using a SpectraMax i3 microplate reader (Molecular Devices, San Jose, CA, USA), and hormone levels were calculated based on standard reference curves.

### 4.10. Statistical Analysis

Germ cell counts, plasma 11-ketotestosterone levels, and gene expression data were analyzed using one-way ANOVA followed by Tukey’s post hoc test for multiple comparisons. For comparisons of *avt* and avt receptor expression between control and pre-spawning fish, unpaired Student’s t-tests were employed. Prior to these analyses, all raw data were assessed for normality using the Shapiro–Wilk test. Outliers were identified and excluded based on the ROUT method. If data did not meet parametric assumptions, non-parametric testing using the Mann–Whitney U test was applied. All statistical analyses were conducted using GraphPad Prism version 8.0 (GraphPad Software Inc., La Jolla, CA, USA).

## 5. Conclusions

The present study provides novel insights into the functional significance of AVT in regulating spermatogenesis in zebrafish. In addition, the findings advance a new model for the role of AVT in regulating zebrafish gonadal function, rooted in the present in vivo results as well as those related to ex vivo actions of AVT on basal and gonadotropin-induced response [18,40]. Chronic AVT exposure leads to the accumulation of undifferentiated spermatogonia, depletion of later-stage germ cells, and reduced sperm output, outcomes driven by alterations in androgen synthesis and FSH receptor activity. These findings suggest that AVT regulates germ cell commitment and maturation primarily through interactions with key hormonal signals such as FSH.

Furthermore, considering AVT’s effects in the presence of LH and the pre-spawning decline in avt and receptor transcripts, our model proposes that AVT is crucial for preparing germ cells at the early spermatogonial stage but becomes dispensable as spermiogenesis approaches. This dynamic regulation ensures proper timing of differentiation, as AVT presence supports early proliferation and differentiation, while its downregulation allows LH-driven progression towards mature spermatozoa.

## Figures and Tables

**Table 1:** Sense and antisense avt primer constructs for ISH.

Probe	Primer Combination	Sequence
Sense avt	T7-FW + RV	5′-TAATACGACTCACTATAGGGTGTCAGACTCTCTGCTGTCT + TAGGCGATGTGTTCAGAAAGG-3′
Antisense avt	FW + T7-RV	5′–TGTCAGACTCTCTGCTGTCT + TAATACGACTCACTATAGGTAGGCGATGTGTTCAGAAAGG-3′

## Data Availability

Data will be made available on request.
